# Application Effect of Combining Image-Text Communication-Based Healthcare Education with Shifting of Attention on Child Patients Undergoing Inguinal Hernia Repair under General Anesthesia

**DOI:** 10.1155/2022/7730158

**Published:** 2022-04-28

**Authors:** Sandong Chen, Wanshun Liang, Shuai Wang, Yingping Jia

**Affiliations:** Department of Anesthesia and Perioperative Medicine, Children's Hospital Affiliated to Zhengzhou University, Zhengzhou 450000, Henan, China

## Abstract

**Objective:**

To analyze the application effect of image-text communication-based healthcare education combined with shifting of attention on child patients undergoing inguinal hernia repair under general anesthesia.

**Methods:**

A total of 110 child patients with inguinal hernia treated in our hospital from January 2020 to January 2022 were selected as the study subjects and divided into the control group (CG, routine intervention measures) and the research group (RG, image-text communication-based healthcare education combined with shifting of attention) according to their preoperative intervention plans, with 55 cases each. After surgery, the child patients' psychological status, crying and shouting situation, and occurrence of complications were evaluated to compare and analyze the intervention effect of the two groups.

**Results:**

The child patients' positive rate and anxiety incidence rate of psychological status evaluation were obviously lower in RG than in CG (*P* < 0.05), and the daily frequency of crying and shouting was significantly lower in RG than in CG (*P* < 0.05); the single time of crying and shouting was significantly shorter in RG than in CG (*P* < 0.05); after surgery, child patients in the two groups had different degrees of infections, subcutaneous emphysema, and scrotal edema, but the total incidence rate of these complications was obviously lower in RG than in CG (*P* < 0.05); after surgery, no significant between-group difference in child patients' FLACC scores immediately after being transferred to the ward was observed (*P* > 0.05), and at postoperative 1 h, 3 h, and 5 h, the FLACC scores of RG were obviously lower than those of CG (*P* < 0.05); and according to the investigation results, the total satisfaction and number of very satisfied parents in RG were greatly higher than those in CG (*P* < 0.05).

**Conclusion:**

Before child patients undergoing inguinal hernia repair under general anesthesia, implementing image-text communication-based healthcare education combined with shifting of attention can effectively improve the child patients' postoperative psychological status and crying and shouting situation and is conducive to preventing postoperative infections, pain, and other complications and promoting postoperative recovery. The combined intervention has potential utility in reducing child patients' high-risk adverse reactions during the perioperative period and ensuring smooth operation, which is generally recognized by the child patients' family members.

## 1. Introduction

Inguinal hernia is a common surgical disease. The overall incidence of inguinal hernia is higher in children due to their unclosed or incomplete closed peritoneal vestigium processus combined with the factors such as crying and constipation [1–3]. Laparoscopic inguinal hernia repair is a relatively effective and safe procedure for the treatment of pediatric inguinal hernias in the clinic. However, the affected children with young age are prone to physiological and psychological traumatic stress reactions after surgery, which can then trigger adverse emotions and increase the risk of complications. Also, children have low tolerance for postoperative pain, which can very easily affect the efficacy of surgery [4–6]. At this stage, the affected children present poor acceptance to the conventional clinical nursing interventions, and their family members are mostly in a state of passive cooperation, and thus, the intervention efficacy is limited. Previous studies have shown that analgesic treatment, which is divided into pharmaceutical type and nonpharmaceutical type, can also alleviate postoperative stress reaction in children. However, common drugs are easy to trigger adverse effects such as nausea, vomiting, headache, and dizziness due to the low specificity, while the nonpharmaceutical measures mainly including shifting of attention are simple and easy, but its single application often shows insufficient intervention intensity [2, 7]. The emerging image-text communication-based healthcare education, which mainly uses pictures and words, is intuitive and efficient and obtains high child acceptance and adaptability, but its application is still somewhat controversial [8]. After analysis, the authors believed that such affected children have immature mind and body, extremely poor self-control, and high dependency on their parents, and therefore, the comprehensive intervention for such children should be based on their parents and implemented in advance. Based on this, the application effect of image-text communication-based healthcare education combined with shifting of attention on child patients undergoing inguinal hernia repair under general anesthesia was explored herein.

## 2. Materials and Methods

### 2.1. Inclusion Criteria

1. The child patients met the clinical diagnosis criteria for pediatric inguinal hernia in the Medical Guidance for Children [1] and were diagnosed after ultrasonic testing and met the surgical indications
2. The child patients received laparoscopic inguinal hernia repair under general anesthesia
3. The child patients had unilateral lesion
4. The child patients' parents had normal communication, cognition, and understanding abilities
5. The child patients' parents understood the study plan, process, and significance and signed the informed consent.

### 2.2. Exclusion Criteria

1. The patients had the history of chronic pain
2. The patients were complicated with other internal medicine diseases
3. The patients had the psychiatric history or cognitive impairment
4. The patients had abnormal coagulation function
5. The patients were less than 2 years old
6. The patients had congenital diseases
7. The patients had important visceral dysfunction
8. The patients were crying persistently and could not receive anesthesia and surgery

### 2.3. Screening and Grouping of Child Patients

A total of 110 child patients with inguinal hernia treated in our hospital from January 2020 to January 2022 were selected as the study subjects and divided into the control group (CG, routine intervention measures) and the research group (RG, image-text communication-based healthcare education combined with shifting of attention) according to their preoperative intervention plans, with 55 cases each. The study met the World Medical Association Declaration of Helsinki (2013) [9].

### 2.4. Methods

After admission, all child patients received relevant physical examinations and laparoscopic inguinal hernia repair; and those in the control group (CG) accepted routine intervention measures, including education to their parents, nutritional support, and pacifying the emotions, and paying attention to position change, incision care, and prevention of intraabdominal hypertension and complications after surgery.

On the basis of CG, image-text communication-based healthcare education combined with shifting of attention was performed in the research group (RG). (1) Preparation: A special nursing intervention team was set up for centralized training, and after the team members passed the exam and reached the standard, relevant preparation works before implementing intervention were completed, including collecting and making textual and graphic files, mastering the psychological features of the affected children, formulating solutions for dealing with crying child patients, determining the education contents, and completing the documents, manuals, pictures, and audio data. (2) A good relationship with the affected children and their family members was established quickly with a kind, friendly, and positive attitude; in particular, more encouragement should be given to the affected children, so that they could increase the confidence in the medical staff, and for those with bad mood, consolation was given or psychological intervention was implemented. (3) Image-text communication: during the perioperative period, color graphic cards were distributed to the child patients mainly for soothing their emotions, and attention was paid to increasing the preoperative intervention intensity and laying the groundwork for postoperative intervention; during the intervention process, the child patients could be accompanied and assisted by their parents (for child patients who could not read on their own, reading could be completed with the assistance of their parents). (4) Image-text communication contents: when setting and selecting the contents, those that were popular and easy to understand, vivid, and interesting and rich in color should be used, e g., the cartoon preferred by children, so as to arouse the children's interest; in case of resentment, crying and shouting, and other bad emotions, interesting images and texts were used to shift the child patients' attention, and at the same time, consolation was performed to relieve their bad emotions, more appreciation and encouragement were given, and one-hour intervention was conducted daily before surgery [10]. (5) After the child patients were awake after surgery, images, texts, audios, videos, and other materials were used to shift their attention, so as to perform intervention before they experienced possible discomforts such as pain, and such intervention could correspond to the preoperative intervention contents for enhancing patients' psychological preparation and confidence to face the discomforts; after that, when the children were awake, movies and music preferred by them were played daily for 2-3 h and 30 min—1 h of image-text communication could be conducted. (6) Healthcare education: in addition to education and intervention implemented to the child patients, the healthcare education to their family members was also strengthened. During the perioperative period, basic knowledge of the disease, daily care precautions, complication prevention, postoperative off-bed activities, emotion soothing, healthy diet, and other knowledge were taught to the patients' family members; moreover, the family members' understanding of image-text communication, healthcare education, and shifting of attention was enhanced, so that they could better cooperate with the nursing personnel; after discharge, guidance and education mainly centered on children's psychological health, diet collocation, exercises, and physical fitness.

### 2.5. Observation Indexes

#### 2.5.1. General Data

The data of child patients' age, weight, height, head circumference, gender, lesion location, surgery time, and ASA grade were recorded.

#### 2.5.2. Psychological Status

According to the subjective evaluation method and scale evaluation method (SAS and SDS) [10], child patients' psychological status was evaluated by psychological tests after surgery. Subjective evaluation method: with the companion of family members, child patients were taken to the treatment room for evaluation of psychological status, the room was kept quiet and comfortable, and the nursing personnel directly communicated with the child patients and their family members and obtained information through sensory perception to judge the nature and extent of the child patients' psychological state. SAS and SDS were the standard scales to measure anxiety and depression in the clinic, which consisted of 20 symptom factors of anxiety and depression, each item was rated on a scale of 1–4 points, and the resulting crude scores were converted into the scale scores, with higher scores indicating more serious anxiety and depression.

#### 2.5.3. Crying and Shouting Situation

The daily frequency and single time of crying and shouting of child patients were observed carefully.

#### 2.5.4. Complication Incidence

The child patients' possible postoperative complications, such as infection, subcutaneous emphysema, and scrotal edema, were observed and recorded.

#### 2.5.5. Pain

The FLACC scale was used to evaluate the child patients' pain degrees, which was suitable for the young children, and mainly included five areas: facial expression, leg movement, activity, cry, and consolability; each item was rated on a scale of 0–2 points, and the total score of the scale was 10 points, with higher scores indicating more serious pain.

#### 2.5.6. Parent Satisfaction

By referring to the Newcastle Satisfaction with Nursing Scales (NSNS) [11], an Investigation Scale for Parent Satisfaction was proposed to investigate parent satisfaction with the intervention process. The total score was 100 points, and the degree of satisfaction was divided into dissatisfied, satisfied, and very satisfied according to the scores. The total satisfaction = (number of satisfied parents + number of very satisfied parents)/total number × 100%.

### 2.6. Statistical Processing

The between-group differences in data were calculated by the software SPSS 22.0, the picture drawing software was GraphPad Prism 7 (GraphPad Software, San Diego, USA), the items included were enumeration data and measurement data, which were expressed by (*n* (%)) and $\left(\bar{x}\pm s\right)$ and examined by the X^2^ test and *t*-test, respectively, and differences were considered statistically significant at *P* < 0.05.

## 3. Results

### 3.1. General Data

Table 1 provides that no statistical between-group differences in patients' general data such as age, weight, height, head circumference, gender, lesion location, surgery time, and ASA grades were observed (*P* > 0.05).

### 3.2. Psychological Status

**Figure 1:**
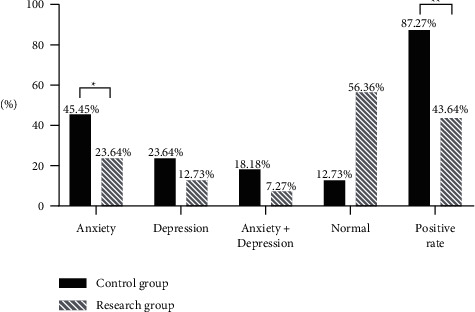
Psychological status evaluation positive rate (*n* = 55)

According to the analysis of statistical data in Figure 1, the positive rate and anxiety incidence rate of psychological status evaluation were significantly lower in RG than in CG (*P* < 0.05), with statistically significant differences.

Note: the horizontal axis indicates the evaluation dimensions, and the vertical axis indicates the percentage (%). In CG, there were 25 cases with anxiety, 13 cases with depression, 10 cases with anxiety and depression, 7 normal cases, and 48 positive cases. In RG, there were 13 cases with anxiety, 7 cases with depression, 4 cases with anxiety and depression, 31 normal cases, and 24 positive cases. ^*∗*^Significant between-group difference in anxiety incidence of psychological status evaluation (*X*2 = 5.790, *P* = 0.016). ^*∗∗*^Significant between-group difference in positive rate of psychological status evaluation (*X*^2^ = 23.1579, *P* < 0.001).

### 3.3. Crying and Shouting Situation

Table 2 provides that the daily frequency of crying and shouting was significantly lower in RG than in CG (*P* < 0.05), and the single time of crying and shouting was obviously shorter in RG than in CG (*P* < 0.05).

### 3.4. Complication Incidence

Table 3 provides that after surgery, the child patients in the two groups had different degrees of infection, subcutaneous emphysema, and scrotal edema, but the total incidence rate of these complications was significantly lower in RG than in CG (*P* < 0.05).

### 3.5. Pain Degree

**Figure 2:**
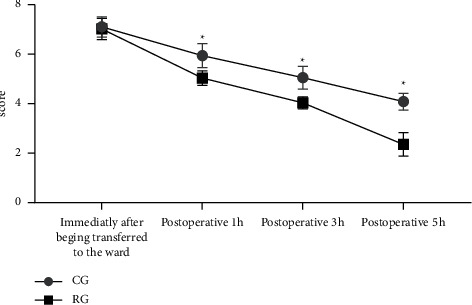
Analysis of patients' postoperative pain $\left(\bar{x}\pm s\right)$

Figure 2 shows that after surgery, no significant between-group difference in child patients' FLACC scores immediately after being transferred to the ward was observed (*P* > 0.05), and at postoperative 1 h, 3 h, and 5 h, the FLACC scores of RG were obviously lower than those of CG (*P* < 0.05).

Note: the horizontal axis indicates the time points, and the vertical axis indicates the score (points). In CG, the FLACC scores immediately after being transferred to the ward and at postoperative 1 h, 3 h, and 5 h were, respectively, (7.10 ± 0.41), (5.94 ± 0.48), (5.05 ± 0.46), and (4.08 ± 0.34). In RG, the FLACC scores immediately after being transferred to the ward and at postoperative 1 h, 3 h, and 5 h were, respectively, (7.02 ± 0.43), (5.03 ± 0.29), (4.03 ± 0.24), and (2.35 ± 0.47).  ^*∗*^ from left to right indicated significant between-group differences in the FLACC scores at postoperative 1 h, 3 h, and 5 h (*t* = 12.254, 14.580, 22.117, *P* all<0.001).

### 3.6. Parent Satisfaction

According to the investigation results, the total parent satisfaction and number of very satisfied parents were significantly higher in RG than in CG (*P* < 0.05), as given in Table 4.

## 4. Discussion

Inguinal hernia is one of the more common surgical diseases in pediatrics, and surgery is the main treatment means. With the continuous development of minimally invasive techniques, laparoscopy has gradually been used in inguinal hernia repair, and the clinical results are relatively satisfactory. But related investigations and studies found that the probability of developing chronic inguinal pain after inguinal hernia repair was about 3–6% and that the preoperative pain was also an important factor leading to postoperative concurrent chronic pain [12]. Postoperative pain will cause the body to abnormally release a series of inflammatory mediators that are painful in response to neuroendocrine stress, which can cause immunoglobulin decline in the affected children, affect postoperative recovery and children's sleep, and cause complications such as incision dehiscence and infection due to crying and shouting. In addition, because of incomplete physical and mental development of children and insufficient parental perception of the disease and other reasons, hernia surgery predisposes affected children to develop negative emotions such as fear, anxiety, and depression, which are not only detrimental to the smooth progress of surgery but also trigger psychological or physiological stress reactions and affect the prognosis [13]. Therefore, implementing effective perioperative intervention measures to child patients undergoing inguinal hernia repair has an active effect on prognosis.

Young children with incomplete perception of the disease may experience extreme fear of surgery as well as postoperative pain, and due to their strong dependency on their parents, they tend to cry and shout hardly after surgery because of pain [14, 15]. By analyzing the clinical features of previous cases, the study performed image-text communication-based healthcare education combined with shifting of attention in the perioperative period to child patients undergoing inguinal hernia repair under general anesthesia, and further education was given to their parents to assist the medical staff in better completing the intervention plan; such child patients were included in RG for comparative analysis with those received routine intervention in CG, and the results were as follows. The positive rate and anxiety incidence rate of psychological status evaluation were obviously lower in RG than in CG (*P* < 0.05); the daily frequency of crying and shouting was significantly lower in RG than in CG (*P* < 0.05), which was consistent with the research report by Damian et al. [16]; the single time of crying and shouting was obviously shorter in RG than in CG (*P* < 0.05); after surgery, child patients in the two groups had different degrees of infection, subcutaneous emphysema, and scrotal edema, but the total incidence rate of these complications was significantly lower in RG than in CG (*P* < 0.05); after surgery, no significant between-group difference in child patients' FLACC scores immediately after being transferred to the ward was observed (*P* > 0.05), and at postoperative 1 h, 3 h, and 5 h, the FLACC scores of RG were obviously lower than those of CG (*P* < 0.05); and according to the investigation results, the total parent satisfaction and number of very satisfied parents were significantly higher in RG than in CG (*P* < 0.05). The study results further confirmed that performing image-text communication-based healthcare education combined with shifting of attention in the perioperative period to child patients undergoing inguinal hernia repair under general anesthesia can effectively pacify their bad mood, improve their psychological status, and lower the frequency and time of crying and shouting; meanwhile, by shifting their attention, their pain sensation can be greatly reduced, which is conducive to preventing postoperative complications and accelerating recovery; hence, the parent satisfaction is higher.

With the image-text communication-based healthcare education combined with shifting of attention, the intervention plan meeting the interest of child patients was formulated to shift their attention and fear about disease, pain, and surgery, reduce their attention to pain, so that they could relax muscles and slow down the breathing frequency, which alleviated their pain sensation to some extent, and combined with the education to the child patients and their parents in the perioperative period, enhanced their confidence in fighting the disease and pain [17–19]. In addition, rich and interesting content, such as pictures, audios, and videos, could promote the interest of the child patients and directly improve the adverse emotions, and the child patients' trust in the medical staff could be enhanced with the positive encouragement of medical staff and their family members and effective communication. When the child patients behaved well, timely affirmation and encouragement could strengthen their positive cooperation with the medical staff, improve the crying and shouting situation, and significantly promote their compliance, which was conducive to lowering the occurrence rate of complications. In addition, because family members are a bridge between the child patients and the medical staff, necessary perioperative healthcare education was conducted to them, so that they could improve their perception of the disease and timely master postoperative-related care measures and simple care skills, avoid anxiety, and convey positive emotions to the child patients. Hence, the parent satisfaction was higher because child patients who were positively impacted could better assist the medical staff in implementing the intervention [20–23]. This intervention modality was simple and easy to execute, with less equipment and funding invested, which was easier to generalize, but the nursing personnel should pay attention to provide careful psychological counseling and effective communication to the child patients during the implementation process; in addition, the extremely special cases were excluded during the pathological screening; thus, there were certain limitations in the study. Future studies should expand the scope of research to analyze the methods that can control the adverse emotions and pain in child patients after surgery in many aspects and optimize the intervention program.

To sum up, before child patients undergoing inguinal hernia repair under general anesthesia, implementing image-text communication-based healthcare education combined with shifting of attention can effectively improve the child patients' postoperative psychological status and crying and shouting situation and is conducive to preventing postoperative infections, pain, and other complications and promoting postoperative recovery. The combined intervention has potential utility in reducing child patients' high-risk adverse reactions during the perioperative period and ensuring smooth operation, which is generally recognized by the child patients' family members.

## Figures and Tables

**Table 1:** Between-group comparison of patients' general data (*n* = 55).

Observation indicator	CG	RG	X^2^/*t*	*P*
Age (years)	6.25 ± 2.10	6.10 ± 2.15	0.370	0.712
Weight (kg)	22.52 ± 2.47	21.70 ± 2.74	1.649	0.102
Height (cm)	117.26 ± 4.78	117.89 ± 5.02	0.674	0.502
Head circumference (cm)	50.14 ± 3.71	50.22 ± 4.10	0.107	0.915
Gender				
Male	43 (78.18)	45 (81.82)	0.227	0.634
Female	12 (21.82)	10 (18.18)		
Lesion location				
Left side	32 (58.18)	30 (54.55)	0.148	0.701
Right side	23 (41.82)	25 (45.45)		
Surgery time (min)	25.91 ± 1.55	26.18 ± 1.62	0.893	0.374
ASA grade				
I	28 (50.91)	31 (56.36)	0.329	0.566
II	27 (49.09)	24 (43.64)		

**Table 2:** Frequency and single time of crying and shouting of child patients in the two groups $\left(\bar{x}\pm s\right)$.

Group	Cases	Frequency of crying and shouting (times/d)	Single time of crying and shouting (min)
CG	55	8.55 ± 2.31	2.37 ± 0.96
RG	55	3.16 ± 1.01	1.05 ± 0.55
*t*		15.855	8.848
*P*		<0.001	<0.001

**Table 3:** Statistics of complications in the two groups.

Group	Infection	Subcutaneous emphysema	Scrotal edema	Total incidence rate
CG (*n* = 55)	8 (14.55)	7 (12.73)	9 (16.36)	24 (43.64)
RG (*n* = 55)	2 (3.64)	1 (1.82)	3 (54.55)	6 (10.91)
*X* ^2^	3.960	4.853	3.367	14.850
*P*	0.047	0.028	0.067	<0.001

**Table 4:** Analysis of parent satisfaction of the two groups (*n* (%)).

Group	Dissatisfied	Satisfied	Very satisfied	Total satisfaction
CG (*n* = 55)	19 (34.55)	17 (30.91)	19 (34.55)	36 (65.45)
RG (*n* = 55)	4 (7.27)	20 (36.36)	31 (56.36)	51 (92.73)
*X* ^2^			5.280	12.369
*P*			0.022	<0.001

## Data Availability

The data used to support the findings of this study are available from the corresponding author upon request.
